# Effects of prenatal mindfulness-based childbirth education on child-bearers’ trajectories of distress: a randomized control trial

**DOI:** 10.1186/s12884-020-03318-8

**Published:** 2020-10-15

**Authors:** Marissa D. Sbrilli, Larissa G. Duncan, Heidemarie K. Laurent

**Affiliations:** 1 Department of Psychology, University of Illinois at Urbana-Champaign, Psychology Building, 603 E Daniel St, Champaign, IL 61820 USA; 2 School of Human Ecology, University of Wisconsin-Madison, Madison, USA; 3 Department of Family Medicine and Community Health, University of Wisconsin-Madison, Madison, USA; 4 Osher Center for Integrative Medicine, University of California, San Francisco (UCSF), San Francisco, USA

**Keywords:** Mindfulness, Childbirth, Postpartum depression, Distress

## Abstract

**Background:**

The perinatal period is a time of immense change, which can be a period of stress and vulnerability for mental health difficulties. Mindfulness-based interventions have shown promise for reducing distress, but further research is needed to identify long-term effects and moderators of mindfulness training in the perinatal period.

**Methods:**

The current study used data from a pilot randomized control trial (RCT) comparing a condensed mindfulness-based childbirth preparation program—the Mind in Labor (MIL)—to treatment as usual (TAU) to examine whether prenatal mindfulness training results in lower distress across the perinatal period, and whether the degree of benefit depends on child-bearers’ initial levels of risk (i.e., depression and anxiety symptoms) and protective (i.e., mindfulness) characteristics. Child-bearers (*N* = 30) in their third trimester were randomized to MIL or TAU and completed assessments of distress—perceived stress, anxiety, and depressive symptoms—at pre-intervention, post-intervention, six-weeks post-birth, and one-year postpartum.

**Results:**

Multilevel modeling of distress trajectories revealed greater decreases from pre-intervention to 12-months postpartum for those in MIL compared to TAU, especially among child-bearers who were higher in anxiety and/or lower in dispositional mindfulness at baseline.

**Conclusions:**

The current study offers preliminary evidence for durable perinatal mental health benefits following a brief mindfulness-based program and suggests further investigation of these effects in larger samples is warranted.

**Trial registration:**

The ClinicalTrials.gov identifier for the study is: NCT02327559. The study was retrospectively registered on June 23, 2014.

## Background

The perinatal period, defined as pregnancy through 12-months postpartum, is a time of immense change in a person’s life [1]. While this time can be met with joy, it is also for many child-bearers a period of stress and vulnerability to mental health difficulties [2]. In particular, a substantial proportion of child-bearers experience one or more components of the “perinatal distress umbrella”—stress, anxiety, and depression—during this period [3], with prevalence estimates of 10–15% across pregnancy and postpartum (e.g., [4, 5]). Given the serious negative impacts on child-bearers’ own health as well as their children’s neurocognitive development [6–8], it is imperative to reduce psychological distress during the perinatal period [9]. Based on findings that different components of perinatal distress can fuel one another across pregnancy-postpartum (e.g., [10]) and that trajectories of distress (as opposed to levels at a single time) matter for mother/infant outcomes [11], it is further critical to identify effective preventive intervention strategies that durably reduce distress across the perinatal period. The present study aimed to address this need by investigating the effects of a brief mindfulness-based intervention during pregnancy on child-bearers’ trajectories of distress through 12-months postpartum.

Programs aimed at building mindfulness—defined as a nonjudgmental and purposeful attention to present-moment experience [12]—may act to produce more healthy ways to relate to stressors by diminishing cognitive and emotional reactivity and providing a resource for coping with stress (see [13]). As proposed by stress and coping theory [14], mindfulness is thought to improve well-being in the face of stress by adaptively changing both primary appraisals of stressors—i.e., decreasing threat perceptions, opening space for re-evaluation as a non-valanced challenge—and secondary appraisals—i.e., increasing perceived capability to meet the challenge through an increased repertoire of accessible coping skills (see [15, 16] for discussion of how mindfulness training supports positive reappraisal and adaptive coping). Indeed, evidence within the Mindfulness-Based Intervention (MBI) literature provides support for a range of benefits including reduced psychological distress in healthy adults [17], improved psychological functioning [13], reduced impact of daily stressors, mitigated psychological distress [18], reductions in anxiety and depression symptoms [19], and the prevention of relapse or recurrence of major depressive disorder [20, 21]. A recent meta-analysis of 142 randomized-controlled trials (RCTs) suggested that MBIs are superior to active controls (and on par with other evidence-based interventions) at post-intervention and follow-up, with effects on depression having the most consistent evidence [22].

During the perinatal period specifically, the evidence is growing that MBIs may aid in mitigating stress and affective symptoms and supports the integration of MBIs into regular pregnancy care in order to reach child-bearers both with and without risk for mental health concerns [23]. Shi and Macbeth’s [23] recent systematic review highlighted the acceptability of MBIs for pregnant people as well as the beneficial effect on different manifestations of perinatal distress—i.e., reductions in stress [24–27], anxiety [24–26, 28–30], and depression [25, 28–33]. Although these components of the perinatal distress umbrella are commonly assessed separately and in parallel within the MBI literature (e.g., [7, 25]), it may be important to explore these both in the aggregate as a multidimensional distress construct and as independent but related drivers of mental health outcomes longitudinally across the perinatal period [3]. It is further worth noting that there is limited evidence for the benefit of antenatal MBIs beyond the immediate post-intervention period; although several studies involving high-risk samples (child-bearers with recurrent major depressive disorder or elevated anxiety) have found sustained effects of an antenatal MBI on symptoms at 3–6 months postpartum [31, 34, 35], other studies have failed to detect significant MBI effects at follow-up (6-week post-intervention – [26]; 3-month postpartum – [29]), and no published work to our knowledge has established effects that endure beyond the first half year postpartum. As discussed above, this may be especially important for evaluating the utility of perinatal prevention strategies for mother/infant health. In sum, despite growing evidence that MBIs can improve perinatal psychological well-being, there are gaps in knowledge of the durability of effects on cumulative distress and/or individual distress components that must be addressed to make strong treatment recommendations.

A promising MBI developed for the perinatal period is the Mindfulness-Based Childbirth and Parenting program (MBCP [28, 36]). MBCP is a formal adaptation of Mindfulness-Based Stress Reduction (MBSR [12, 37]) targeted at the needs of pregnant people and partners approaching childbirth. Alongside basic training in mindfulness through didactic content and guided meditation practices, MBCP offers content related to managing childbirth- and parenting-related stress and pain and building supportive relationships across the transition to parenthood [28]. Empirical evidence supports the intended impact and process of MBCP through increases in mindfulness (especially the nonreactivity facet) and positive affective states and decreases in pregnancy anxiety [28], stress, and depression [38]. A briefer adaptation of MBCP (4 rather than 9 weeks) also was shown to yield significant improvement in child-bearers’ stress, depression, and anxiety [39].

In further efforts to increase feasibility and accessibility of this intervention, Duncan and colleagues [33] created a condensed, 2.5-day weekend childbirth preparation workshop based on MBCP called The Mind in Labor (MIL). In the first report on their pilot RCT of MIL effects, Duncan et al. found greater improvements in childbirth self-efficacy and depression symptoms associated with MIL compared to treatment as usual (TAU) at 6-weeks post-birth. This offers a promising starting point for evaluating potential longer-term effects of this brief intervention.

In addition to evaluating the overall efficacy of interventions, it is important to investigate moderators of intervention outcomes (i.e., factors that predict differential responses to the intervention) to determine who may optimally benefit from such programs and inform personalized medicine [40]. Recent work has underlined the importance of examining moderators of MBIs’ impact in order to move toward a personalized approach to intervention matching based on baseline patient characteristics [41]. Overall, it appears that those who begin an intervention with the greatest needs are likely to show the greatest improvement. In line with meta-analytic evidence in the (non-MBI) intervention literature (e.g., [42, 43], several studies have reported greater benefit following MBSR [12, 37] among individuals with higher starting levels of distress (anxiety symptom severity – [44], depression symptoms and anxiety sensitivity – [45]). Baseline levels of dispositional mindfulness have also been shown to moderate outcomes following MBIs, with the preponderance of evidence favoring greater benefit among participants with higher pre-intervention mindfulness ([46]; though see also [47] for mixed findings including moderated effects in the opposite direction). Although we might expect, based on these indications, that pregnant people experiencing higher distress and/or endorsing higher levels of mindfulness qualities would gain the most from a program like MIL, there is no prior research to our knowledge addressing individual difference moderators of MBI effects in a perinatal population.

## The current study

Taken together, the research reviewed above provides growing evidence for the benefit of MBIs in pregnancy to support maternal well-being [48], while also highlighting a need for further research to elucidate the nature and durability of benefits. In particular, further investigation is required to establish whether mindfulness training predicts lasting differences in trajectories of often comorbid manifestations of perinatal distress, as well as to identify baseline characteristics that moderate these effects. The current study aimed to address this need by examining the following research questions: 1) Is participation in MIL versus TAU associated with different trajectories of distress—child-bearers’ perceived stress, anxiety, and/or depressive symptoms—across pregnancy and postpartum? 2) Does the symptom level and/or dispositional mindfulness of child-bearers’ at baseline moderate the impact of childbirth education class assignment on distress trajectories? Based on both the prior empirical evidence and predictions based on stress and coping theory, we hypothesized that participation in MIL would be associated with greater reductions in distress—evidenced by decreasing slopes and lower final distress levels—compared to TAU. We further predicted moderated effects such that child-bearers with higher levels of depression or anxiety symptom severity and/or higher levels of dispositional mindfulness at baseline would display the greatest improvements in MIL compared to TAU. Although primary hypotheses involved cumulative distress outcomes as a composite of stress, anxiety, and depressive symptoms, secondary analyses examined each outcome measure separately to determine whether effects appeared to apply specifically to a particular dimension of distress.

## Method

### Participants

**Fig. 1:**
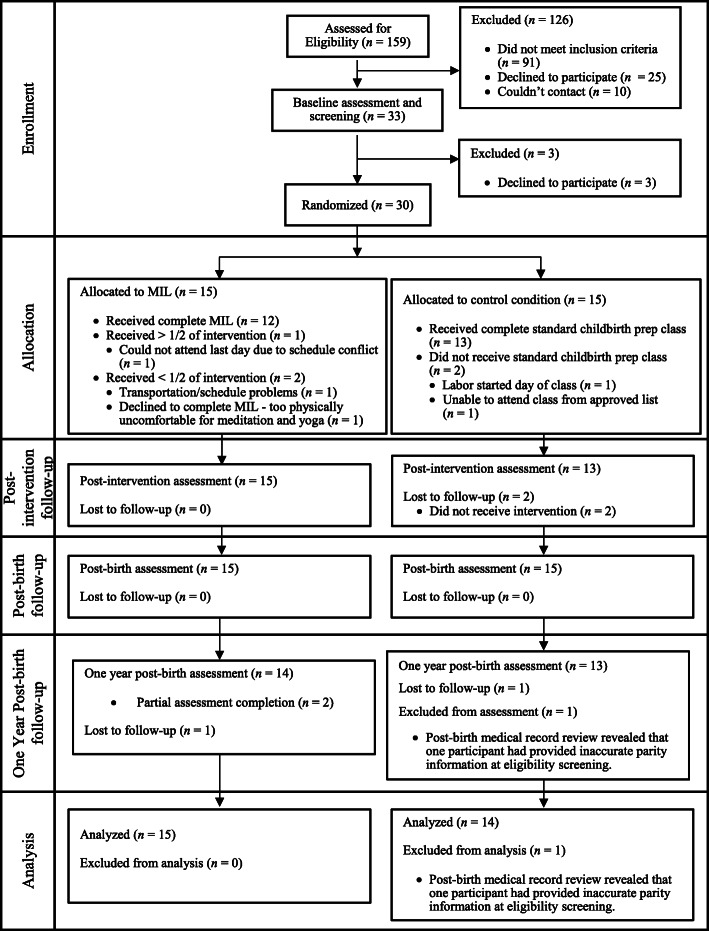
Participant flow chart. *Note*. Figure adapted from [33]. MIL = Mind in Labor: Working with Pain in Childbirth

This study utilized data from the pilot RCT comparing MIL to TAU first described by Duncan and colleagues [33]. Participants were recruited through: 1) provider referral, 2) the internet and word of mouth (e.g., parenting message boards, Google advertisements, and list-serves), and 3) posted flyers targeting pregnant people with fear of childbirth. The participant sample included 30 nulliparous child-bearers with low-risk, healthy, single baby pregnancies in their third trimester who planned to give birth in a hospital and were willing to be randomized to either condition. Exclusion criteria included any previous experience in meditation or yoga (barring prenatal yoga, which did not warrant exclusion), involvement in a separate mind/body childbirth class, planned homebirth or other non-hospital setting, or a planned Cesarean delivery. The sample was 59% White (*n* = 17), 17% Asian (*n* = 5), 14% Multiracial (*n* = 4), 7% Black/African American (*n* = 2), and 3% American Indian/Alaska Native (*n* = 1). Regarding ethnicity, 18% of the participants were Hispanic/Latinx (*n* = 5, missing = 1). One participant in MIL and two participants in TAU completed childbirth education classes independently (i.e., without a birth partner). Below median household income for the area (<$90 k) was reported by 55% of the participants (*n* = 16) and 10% of the sample reported a household income of less than $10,000 a year (*n* = 3; See [33] for details). The sample was relatively low risk regarding mental health (see Table 1) with baseline clinical characteristics similar to those of a universal sample versus a selected population of child-bearers at higher risk for mental health issues (e.g., [30]). The current study adheres to CONSORT guidelines; see Fig. 1 for the CONSORT flow chart of study recruitment and participation.

**Table 1:** Baseline Mental Health: Means and Clinical Cut-Offs

	CES-D	STAIT	PSS	FFMQ
Clinical Cut-Off	≥16	≥40	N/A	N/A
*M(SD)*	9.66 (8.05)	36.07 (8.62)	15.34 (6.25)	3.47 (0.35)
N (%) above Clinical Cut-Off	6 (20%)	10 (34%)	N/A	N/A

*Note.* None of the baseline mean scores were above clinical cut-off for the scales that provide them (i.e., CES-D and STAIT). *CES-D* = Center for Epidemiologic Studies Depression, *FFMQ* = Five Facet Mindfulness Questionnaire, *PSS* = Perceived Stress Scale, *STAIT* = State Trait Anxiety Inventory – Trait

### Procedures

All study procedures were approved by the University of California, San Francisco (UCSF) Committee for Human Research (institutional review board), and signed informed consent was obtained from all participants. Participants were randomized to either MIL (*n* = 15) or TAU (*n* = 15) using a pre-programmed computer database. Self-report measures were completed online at four time points: time 1 (T1) was the third trimester baseline (immediately pre-intervention and pre-randomization), time 2 (T2) was the week immediately following the intervention (post-intervention but prior to birth), time 3 (T3) was the postpartum follow-up (approximately 6 weeks post-birth), and time 4 (T4) was 1 to 2 years post-birth. Due to the timing of received project funding for long-term follow-up, T4 assessment timing varied such that earlier cohorts completed T4 up to 2 years post-birth while later cohorts completed T4 at 1 year post-birth. Participants completed the T4 assessment on average 1.79 years post-birth (*M* = 93.08 weeks, *SD* = 0.17 years, range = 1.47–2.20 years. All eligibility screening and assessment was conducted through an online survey software (see [33] for further details of compensation and time period of data collection). The current study was submitted in fulfillment of the first author’s master’s thesis (see [49]).

### Interventions

#### Mind in labor (MIL): working with pain in childbirth

As described above, MIL is a short, time-intensive weekend (2.5 day) childbirth education program adapted from MBCP for pregnant people and their partners that integrates mindfulness strategies for coping with pain and fear with formal mindfulness meditation for a total of 18 h of mindfulness training. MIL was conducted by certified MBCP instructors and facilitated by the developer of MBCP. MIL teaches mindfulness strategies for coping with labor-related pain and fear through interactive, experiential exercises, alongside didactic instruction on how mindfulness may be brought to bear on childbirth preparation (e.g., birth physiology) and parenting an infant. Handouts and CDs/ mp3s of guided mindfulness meditations were provided to participants for optional practice following MIL. Participants’ birthing support partners were invited to attend MIL; most partners attended, with one child-bearer participating in MIL independently. There was no cost to participants for the MIL program (see [33] for further details).

#### Treatment as usual

TAU was an active control condition in which participants were able to choose a standard childbirth education class from a list of approved community resources or a class suggested by a participant that received approval from study staff. These were typical childbirth education program options that featured no mind/body focus, mindfulness meditation, or yoga. Content was determined by reaching out to providers and inquiring about any mind/body or stress-related subject matter. Participants could also request a non-listed class in the case that a listed course was not convenient given their location/schedule, and it was evaluated using the same procedures to create the approved list. Participants were given up to $200 to cover the tuition for their approved childbirth education program.

### Measures

#### Center for Epidemiologic Studies Depression Scale

Depression was measured by the Center for Epidemiologic Studies Depression Scale (CES-D [50]) at T1-T4. Participants rated their experience of various depression symptoms over the past week on the widely-used, 20-item self-report measure using a scale from 0 (“*Rarely or none of the time*”) to 3 (“*Most or all of the time*”). A score of ≥16 is used to indicate clinical levels of depression. The analyses in the current study utilize CES-D scores from baseline through one-year post-birth (*α* = .80 to .89).

#### Spielberger state-trait anxiety inventory – trait

Anxiety was measured by the Spielberger State-Trait Anxiety Inventory – Trait (STAIT [51]) at T1-T4. The scale prompted participants to rate “how you generally feel” for 20 anxiety symptoms on a scale from 1 (“*Almost never*”) to 4 (“*Almost always*”). A score of ≥40 is used to indicate clinical levels of trait anxiety. The data from pre-intervention through one-year postpartum reflected good internal consistency (*α* = .90 to .95).

#### Perceived stress scale

Perceived stress was measured by the Perceived Stress Scale (PSS [52]) at T1-T4. The PSS is a widely used measure of global stress perception—i.e., the degree to which a person perceives life events to be stressful. Participants reported the frequency of 10 stress-related thoughts and feelings in the past month on a scale from 0 (“*Never*”) to 4 (“*Very Often*”). This scale is used as a continuous variable, with no set clinical cut-off, where higher scores indicate higher levels of stress. Participants’ scores on the PSS were collected at baseline through one-year postpartum (*α* = .87 to .94).

#### Five facet mindfulness questionnaire

Mindfulness was measured by the Five Facet Mindfulness Questionnaire (FFMQ [53]) at T1. The FFMQ is a 39-item measure that assesses five dimensions of mindfulness identified through a factor analysis of existing mindfulness scales, which include observing, describing, acting with awareness, nonjudging of inner experience, and nonreactivity to inner experience. Items are rated on a scale from 1 (“*Never or very rarely true*”) to 5 (“*Very often or always true*”) and were averaged to obtain a total mindfulness score (*α* = .88); to test proposed moderated effects, only the baseline (T1) score was used in analyses.

### Data analysis plan

We employed an intent-to-treat analysis in which all eligible and randomized participants were included in the analyses with the sample of *N* = 29 (*n* = 14 TAU and *n* = 15 MIL) regardless of their degree of participation in the study. One participant in the control condition was excluded from analyses due to incorrect report of parity in the eligibility assessment (see Fig. 1). To address effects on child-bearers’ total distress, a distress composite score of the STAIT, CES-D, and PSS was created at each wave. This combination was found to be justified by correlations among the measures (*r* = .56–.90; see Table 2) and internal reliability of the composite score, which was created by standardizing and then averaging the scores (similar to composites made for other investigations of internalizing symptoms in the perinatal period [54]).

**Table 2:** Correlations of Distress Components and Mindfulness

	*STAIT_T2*	*STAIT_T3*	*STAIT_T4*	*PSS_T1*	*PSS_T2*	*PSS_T3*	*PSS_T4*	*CESD_T1*	*CESD_T2*	*CESD_T3*	*CESD_T4*	*FFMQ_T1*
*STAIT_T1*	0.631^***^	0.334	0.503^**^	0.676^***^	0.356	0.069	0.385	0.565^**^	0.332	−0.095	0.330	−0.678^***^
*STAIT_T2*		0.577^**^	0.794^***^	0.427^*^	0.759^***^	0.186	0.654^***^	0.413^*^	0.695^***^	0.259	0.550^**^	−0.404^*^
*STAIT_T3*			0.714^***^	0.140	0.750^***^	0.643^***^	0.745^***^	0.266	0.652^***^	0.712^***^	0.625^***^	−0.183
*STAIT_T4*				0.227	0.692^***^	0.182	0.904^***^	0.266	0.714^***^	0.426^*^	0.836^***^	−0.367
*PSS_T1*					0.397^*^	−0.185	0.127	0.820^***^	0.303	−0.202	0.119	−0.631^***^
*PSS_T2*						0.454^*^	0.668^***^	0.459^*^	0.806^***^	0.593^**^	0.578^**^	−0.353
*PSS_T3*							0.363	−0.089	0.289	0.750^***^	0.174	0.002
*PSS_T4*								0.259	0.710^***^	0.579^**^	0.850^***^	−0.266
*CESD_T1*									0.510^**^	0.047	0.298	−0.567^**^
*CESD_T2*										0.493^**^	0.716^***^	−0.181
*CESD_T3*											0.487^*^	0.061
*CESD_T4*												−0.267
*FFMQ_T1*												

Computed correlation used pearson-method with pairwise-deletion

*Note. CESD* = Center for Epidemiologic Studies Depression, *FFMQ* = Five Facet Mindfulness Questionnaire, *PSS* = Perceived Stress Scale, *STAIT* = State Trait Anxiety Inventory – Trait

*indicates *p* < .05. ** indicates *p* < .01. *** indicates *p* < .001

We used multilevel modeling in HLM to examine trajectories of pregnant people’s distress across time and to test proposed differences by intervention group. As outlined above, distress outcomes were operationalized by the composite score for primary analyses and broken down by specific scales (i.e., CES-D, STAIT, PSS) in secondary analyses. Level 1 modeled each child-bearer’s distress trajectory with an intercept and linear slope; the latter was centered at the final (T4) assessment so that intercepts represented final levels of distress at 12- to 24-months postpartum. Level 2 modeled between-person differences in distress trajectories that could be explained by intervention condition (testing study question/hypothesis 1), as well as interactions of intervention condition with baseline levels of symptoms or mindfulness (testing study question/hypothesis 2).

## Results

First a baseline multilevel model containing no explanatory predictors was fit to describe child-bearers’ distress trajectories across pregnancy and postpartum follow-up. Although the linear slope was not significant, indicating that distress levels did not consistently change over time in the sample as a whole, significant between-person variability in both intercepts (χ^2^[27] = 91.86, *p* < .001) and slopes (χ^2^[27] = 45.23, *p* = .015) suggested heterogeneity in course of distress. That is, some child-bearers experienced increasing distress and others decreasing distress from the third trimester pregnancy through 12- to 24-months postpartum, supporting the addition of predictors to explain differences in child-bearers’ distress slopes and ending levels (intercepts).

The first explanatory model tested overall differences in distress trajectories between child-bearers who participated in the MIL intervention and those in the TAU condition by entering the dummy-coded variable indicating MIL participation as a predictor of distress intercepts and slopes (see Table 3, panel A for model results). Although no significant effects based on the *p* < .05 threshold were found, there was a trend for MIL participants to show lower (decreasing) distress slopes, and including the condition predictor explained 23% of the baseline variance in distress slopes. In a secondary step, effects on the individual symptom components of the distress composite—i.e., CES-D depression, STAIT anxiety, and PSS perceived stress—were examined. The effect of intervention condition on the depression symptom slope was significant (γ = −.16, *p* = .041; no other significant effects), suggesting this was the component of child-bearers’ distress most influenced by MIL participation.

**Table 3:** Primary Model Results: Effect of Intervention Condition on Child-Bearers’ Distress Trajectories and Moderation by Baseline Characteristics

Variable	***γ***	***p***
**A. Main Effects**		
Distress Intercept (predicted T4 level)	.045	.816
MIL Participation	−.250	.229
Distress Slope (T1–4 change)	.012	.871
MIL Participation	−.123	.098
**B. Moderated Effects – Baseline Symptoms**		
Distress Intercept (predicted T4 level)	.012	.945
MIL Participation	−.261	.162
T1 Anxiety	.641	.009
MIL x T1 Anxiety	−.584	.004
Distress Slope (T1–4 change)	.011	.858
MIL Participation	−.106	.101
T1 Anxiety	−.040	.553
MIL x T1 Anxiety	−.172	.005
**C. Moderated Effects – Baseline Mindfulness**		
Distress Intercept (predicted T4 level)	.018	.908
MIL Participation	−.300	.123
T1 Mindfulness	−.796	.020
MIL x T1 Anxiety	.787	.006
Distress Slope (T1–4 change)	.003	.957
MIL Participation	−.103	.111
T1 Mindfulness	−.120	.231
MIL x T1 Mindfulness	.316	.001

*Note. γ* = standardized coefficient from HLM model; MIL = Mind in Labor condition (vs. TAU); Distress = composite of Center for Epidemiologic Studies Depression (CES-D), State Trait Anxiety Inventory – Trait (STAIT), and Perceived Stress Scale (PSS) scores; Anxiety = STAIT; Mindfulness = Five Facet Mindfulness Questionnaire (FFMQ) total

**Fig. 2:**
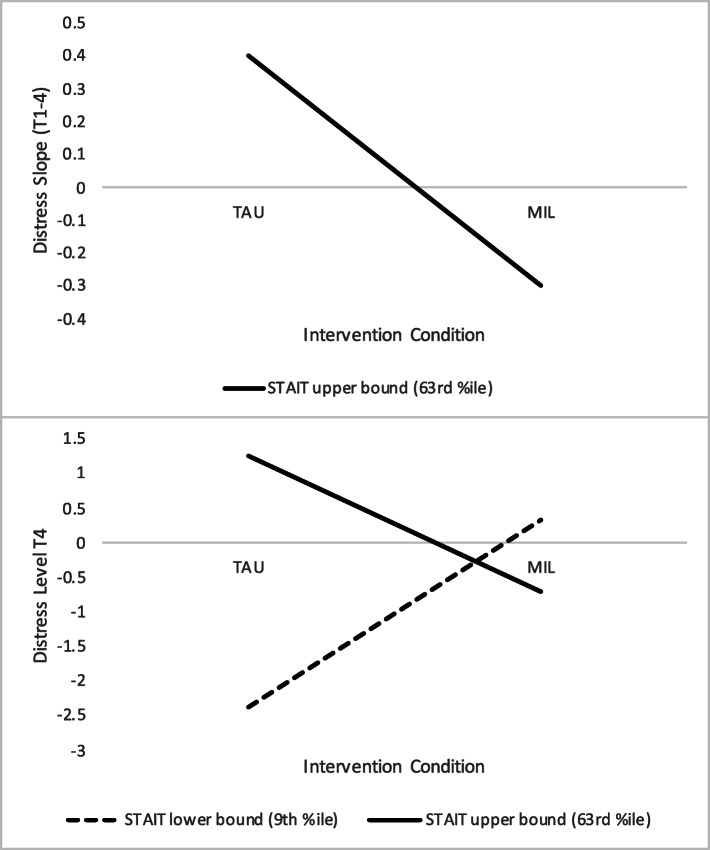
Effects of MIL participation on child-bearers’ distress slopes and ending levels at region of significance boundaries for baseline anxiety. *Note*. STAIT = Spielberger State-Trait Anxiety Inventory – Trait; MIL = Mind in Labor: Working with Pain in Childbirth; TAU = treatment as usual

The next set of explanatory models tested moderated effects of the MIL intervention—that is, whether degree of mental health benefits depended on child-bearers’ starting levels of risk and promotive factors—by adding time 1 symptom or mindfulness scores and interactions with the MIL participation variable to the above model. Significant intervention x time 1 STAIT anxiety interaction effects on both distress intercepts and slopes were observed (see Table 3, panel B). To decompose these interactions, an online calculator [55] was used to determine the region/s of significance, or range/s of the moderator (anxiety) at which the focal predictor (MIL participation) had a significant effect. Based on these calculations, MIL participation resulted in lower (decreasing) distress slopes and lower ending distress levels for child-bearers with moderate to high levels of prenatal anxiety (STAIT scores >63rd percentile). For those with very low starting levels of anxiety (STAIT scores <9th percentile), MIL participation was associated with higher ending distress levels relative to TAU, though their ending distress levels did not differ from those of higher anxiety MIL participants (and the lower boundary value at which a positive effect on distress slopes would be expected was beyond the range of possible STAIT values; see Fig. 2 for illustration). This model explained 32% of the variance in child-bearers’ distress intercept and 58% of the variance in their slopes. Secondary analyses involving specific distress measures showed that, while significant moderated effects applied to all aspects of child-bearers’ distress, they were particularly large/strong for the anxiety (STAIT) outcome.

**Fig. 3:**
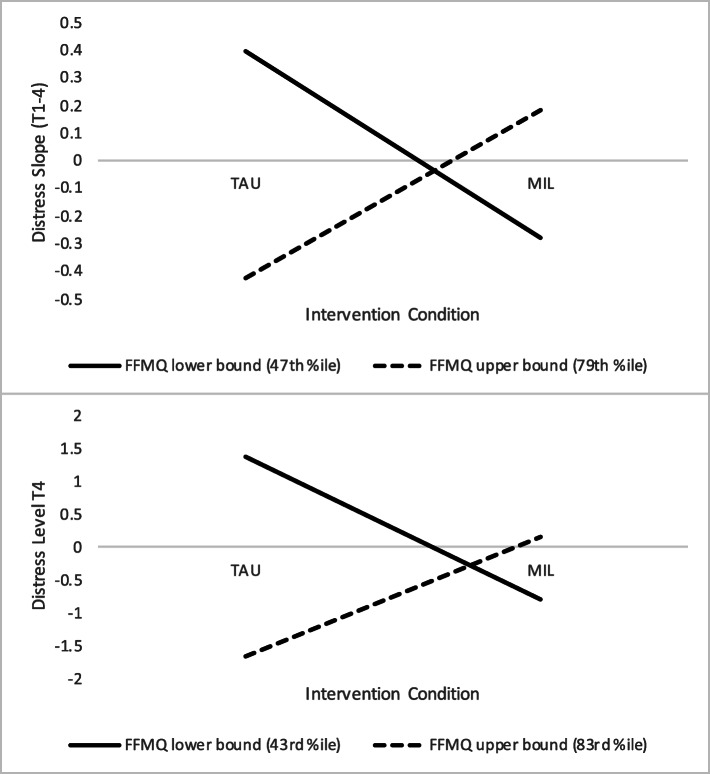
Effects of MIL participation on child-bearers’ distress slopes and ending levels at region of significance boundaries for baseline mindfulness. *Note*. FFMQ = Five Facet Mindfulness Questionnaire; MIL = Mind in Labor: Working with Pain in Childbirth; TAU = treatment as usual

The model including starting levels of dispositional mindfulness as a moderator also revealed significant interaction effects on child-bearers’ distress intercepts and slopes (see Table 3, panel C). Again, region/s of significance testing was used to interpret these effects. Results of these calculations showed that for child-bearers with moderate to low starting levels of mindfulness (< 43rd percentile for intercept effect, < 47th percentile for slope effect), MIL participation resulted in lower (decreasing) distress slopes and lower ending distress levels. On the other side, child-bearers with very high starting levels of reported mindfulness (> 83rd percentile for intercept effect, > 79th percentile for slope effect) who participated in MIL showed higher distress slopes and ending distress levels compared to TAU, though again this did not result in elevated distress relative to lower mindfulness MIL participants (see Fig. 3). This model explained 41% of the variance in child-bearers’ distress intercepts and 90% of the variance in their slopes. Secondary analyses revealed similarly sized moderated effects for each distress outcome under consideration.

Overall, results of the above models support the hypothesized benefit of prenatal mindfulness training through MIL for child-bearers’ perinatal distress through the first to second year postpartum, with the most marked benefit expected for pregnant people higher in anxiety and/or lower in mindfulness. The opposite trend observed in child-bearers who began the intervention with particularly low anxiety/high mindfulness apparently led to an equalization of distress between lower- and higher-risk participants following the MIL intervention.

## Discussion

This study provides preliminary evidence that a brief mindfulness-based childbirth preparation program during pregnancy can shift child-bearers’ trajectories of distress through one-year post-birth. In particular, we found that participation in MIL (compared to TAU) predicted a decrease in the depressive symptom component of perinatal distress, with trend-level effects on an overall distress aggregate. We further found that those with the greatest mental health needs—i.e., pregnant people with higher anxiety and/or lower mindfulness at baseline—received the most benefit, with significant reductions in distress slopes and lower ending levels of postpartum distress compared to their TAU counterparts. Processes driving these findings and implications for perinatal mental health promotion are considered below.

We found partial support for the hypothesis that MIL participation would impact perinatal distress trajectories in the sample as a whole. Although the main effect of intervention condition on the composite measure of perinatal distress did not reach significance, there was a significant effect on the depressive symptom component such that MIL participants showed a greater decrease in symptoms from pregnancy through 12- to 24-months postpartum. This is in line with a recent meta-analysis of MBI RCTs showing the most consistent evidence supported benefits for depression outcomes [22]. It may be that mindfulness training is particularly helpful in changing the self-limiting beliefs and behaviors tapped by depression measures, and MBI mechanisms with the most consistent meta-analytic support—i.e., reactivity to experience and rumination—are known to play a key role in precipitating and maintaining depression [56]. Another explanation for the preferential effect on this component in the current study may have to do with the framing of measures used; of the three distress scales, the depressive symptom measure assessed the most recent time period (past week as opposed to longer/more general time frames for anxiety and stress measures) and may thus have been best poised to capture changes over time. Further longitudinal research using a variety of intermediate and longer-term outcome measures will be needed to probe effects of MBIs on different components of the perinatal distress umbrella and mechanistic processes driving these across childbirth and postpartum development such as cognitive biases, rumination, and avoidance.

In addition to main effects, we were interested in baseline individual difference characteristics that might blunt or boost effects of antenatal mindfulness training. In line with hypotheses, child-bearers with higher initial levels of anxiety symptoms showed a greater benefit of MIL—decreasing distress slopes through one- to two-years postpartum and lower ending levels of distress. This finding is consistent with prior work showing that people with higher starting levels of psychological symptoms derive the greatest benefit from MBIs [43, 57], and with the idea that mindfulness may function to interrupt the transdiagnostic processes that maintain anxiety and other distress-related symptoms [56]. Those with more trait-like and/or pronounced internalizing distress may be especially prone to the automatic reactive processes targeted by mindfulness principles and practices. Mindfulness training brings greater awareness of such automatic reactivity and provides a new approach to the person-experience relationship that is present focused, decentered, accepting, and nonjudgmental [58]. Mindfulness-related gains in flexible appraisal, acceptance of experiences, and positive affect in turn support adaptive coping with perinatal stressors and ultimately mother and child health [15]. By altering the course of at-risk child-bearers’ distress, MIL thus has the potential to exert cascading effects on stress resilience across generations.

Those in MIL with very low initial levels of anxiety showed ending distress that was similar to that of higher anxiety MIL participants, which represented higher ending distress compared to their TAU counterparts with initial low anxiety. This pattern may reflect the increased awareness that comes with beginning engagement with mindfulness practice. That is, individuals who are living on autopilot may simply not be aware of much of their internal experience and report lower symptoms. Being guided into greater awareness of what is going on with oneself somatically, and with one’s thoughts and emotions, may in itself bring about more reporting of symptoms for those who had little awareness prior to an MBI. It is possible that a longer period of follow up—particularly with those who continue in their mindfulness practice—would show an increase in wellbeing and lower symptoms following this initial dip. That is, the benefits of mindfulness practice for child-bearers who start with negligible symptoms may require a longer time scale of sustained practice to observe, and future research should examine this longer term trajectory.

Examining both a composite distress score and distinct distress components provided preliminary evidence that benefits of mindfulness intervention hold to some extent across these three related domains of perinatal mental health, while also highlighting some differential effects across domains. The present findings suggest it may important to consider each of these to fully appreciate impacts of mindfulness training; for example, if only subjective stress or anxiety had been assessed, we would have concluded that MIL participation did not significantly affect child-bearers’ long-term well-being. Similarly, if anxiety symptoms had not been examined, insight into pre-existing characteristics that may make MIL most beneficial would have been limited. Clearly, replication in larger samples will be needed to determine the robustness of these particular effects, but the current study supports the value of considering perinatal distress as both a multidimensional composite (to gauge cumulative effects on mother/child well-being) and a set of individual domains (to interrogate syndrome-specific main and moderated effects).

We also found that baseline levels of dispositional mindfulness moderated the effect of mindfulness training, though in the opposite direction of our original hypothesis; that is, child-bearers with *lower* (rather than higher) starting levels of mindfulness showed the greatest reduction in distress following MIL. Decomposition of these effects revealed a homogenization of distress following MIL between those who started with higher and lower self-reported mindfulness, suggesting that improvements in typical distress measures are especially likely for people who do not already see themselves as mindful and for whom this represents a new approach to the person-experience relationship. For those already primed to think of themselves as mindful, what happens during and following such an intervention may be less associated with an increase in reported well-being; instead, the training may instigate more of a reflective process that involves accessing and reporting levels of distress roughly equivalent to those who began in greater distress and saw improvements. This pattern in consistent with that proposed and partially supported by Gawrysiak and colleagues [47] in which lower baseline mindfulness allows more “room to grow” from an intervention. An explanation advanced by these authors for divergence from previous work (i.e., [46])—that the direction/strength of moderation may depend on the whether the mindfulness measure focuses on attitudinal qualities like acceptance vs. attentional control—may be applicable. The measure used in the present study included key attitudinal facets of mindfulness (nonreactivity, nonjudgment) that overlap with subjective distress. Thus, consistent with the above moderated effect involving anxiety, pregnant people who entered MIL more prone to emotional reactivity were likely able to show the most marked improvements as they learned to approach stressors in a less reactive, more accepting way.

The effects detected here carry important implications for community-based prevention in that a workshop like MIL is likely to be more acceptable and accessible than common alternative interventions for the child-bearers who could benefit the most. That is, pregnant people who are more anxious but who (for reasons of stigma or cost) do not seek mental health treatment could gain valuable tools for improving their well-being through a relatively low-cost, low-commitment workshop. Similarly, child-bearers who do not already endorse mindfulness-related values and dispositions (and who may be deterred by a typical 8 + −week MBI) could become more open to mindfulness-based and other healing practices through the benefit experienced in the workshop. This is not to dismiss the value of the extended training offered by more traditional MBIs (e.g., potential benefits of the foci on developing a regular formal meditation practice and mindful parenting skills offered in the full MBCP but not the abbreviated MIL), but it does suggest that even a relatively brief exposure to mindfulness can bring about sustained benefits during a critical transitional period. These results support further investigation of condensed, intensive mindfulness intervention effects. Future studies should explore characteristics of mindfulness training needed to initiate and sustain distress reduction for different pregnant people. As part of a personalized intervention approach, it will be important to determine how the intensity and emphasis of intervention may be differentially beneficial depending on pregnant people’s particular experiences (e.g., prior births, positive/negative contact with healthcare systems) and circumstances (e.g., family and community support, cultural and socioeconomic context).

Alongside multiple strengths of this study, including the RCT design and long-term follow-up period, there are limitations that should be considered. As a modestly scaled pilot study, this work is best framed as providing an essential foundation for a larger RCT to develop evidence-based recommendations. Small sample size restricts power for detecting effects, particularly smaller-sized effects such as would be expected for interactions, and both null and significant findings in the present study should be considered preliminary. Due to the schedule of funding for this project, the timing of the final assessment was varied across participants (between one and a half to 2 years postpartum), which warrants consideration. The trajectory approach taken here, which highlights slopes of changes over time rather than change between distinct timepoints mitigates the issue this may pose in interpreting results. Future studies should include additional assessments beyond 2 years to examine trajectories into childhood. Conclusions are necessarily limited by the particular measures used, which offered a multidimensional but far from comprehensive measure of the perinatal distress umbrella. It may be worthwhile in future research to include measures of perinatal-specific psychological and somatic symptoms, as well as measures that cover different time frames from immediate state affect—perhaps using experience sampling methods—to more stable trait-like features of psychological functioning.

Efforts to generalize the present findings should further be tempered by acknowledgment that the sample comprised a relatively low-risk group of child-bearers in terms of mental health, and further investigation in clinical samples is warranted to establish benefits at more severe ranges of psychopathology. Additionally, the current study is limited due to the predominantly White sample. Given that African Americans are less likely to receive mental health services than other racial groups and experience persistent risk factors including marginalization and race-based stressors across the lifespan [59], it is crucial that future research examine the needs of child-bearers of color specifically and the extent to which the current findings can be generalized to ethnically and racially diverse populations. While meta-analytic evidence suggests that MBIs and other contextual approaches may be well suited to meet the needs of African Americans [60], more research is needed to inform the efficacy of MBIs specifically for child-bearers of color. Finally, while the TAU condition offered a credible and pragmatic comparison for gauging added benefits of mindfulness training, a more stringent test of MIL effects could involve comparison with other validated treatments for perinatal distress, including both mindfulness-based (e.g., MBCT-PD) and non-mindfulness-based (e.g., interpersonal psychotherapy) interventions.

## Conclusions

Limitations notwithstanding, the present investigation provides preliminary support for the long-term effects of a condensed mindfulness training program on child-bearers’ perinatal distress, particularly for those at greatest risk of stress-related mental health problems. Our findings highlight the value of continuing to validate and disseminate such programs, given barriers to completing more extensive interventions and the importance of promoting maternal wellbeing during this period. As such, this study represents an important step in the larger progression of building an evidence base for mindfulness-based interventions in the prevention of common mental health difficulties that child-bearers face in the powerful yet vulnerable time of pregnancy and postpartum.

## Data Availability

The datasets used and/or analyzed during the current study are available from LGD on reasonable request.

## Abbreviations

CES-D: Center for Epidemiologic Studies Depression Scale
FFMQ: Five Facet Mindfulness Questionnaire
MBCP: Mindfulness-Based Childbirth and Parenting
MBI: Mindfulness-Based Intervention
MBSR: Mindfulness-Based Stress Reduction
MIL: Mind in Labor: Working with Pain in Childbirth
PSS: Perceived Stress Scale
RCT: Randomized controlled trial
STAIT: Spielberger State-Trait Anxiety Inventory – Trait
T1: Time 1 (baseline/pre-intervention)
T2: Time 2 (post-intervention but prior to birth)
T3: Time 3 (six weeks post-birth/postpartum follow-up)
T4: Time 4 (one to two years post-birth)
TAU: Treatment as usual
UCSF: University of California, San Francisco
